# Ticking and talking in the brainstem satiety centre: Circadian timekeeping and interactions in the diet-sensitive clock of the dorsal vagal complex

**DOI:** 10.3389/fphys.2022.931167

**Published:** 2022-09-02

**Authors:** Lukasz Chrobok, Jake Ahern, Hugh D. Piggins

**Affiliations:** Faculty of Life Sciences, School of Physiology, Pharmacology and Neuroscience, University of Bristol, Bristol, United Kingdom

**Keywords:** circadian clock, multi-clock model, dorsal vagal complex, extra-SCN oscillators, feeding pattern

## Abstract

The dorsal vagal complex (DVC) is a key hub for integrating blood-borne, central, and vagal ascending signals that convey important information on metabolic and homeostatic state. Research implicates the DVC in the termination of food intake and the transition to satiety, and consequently it is considered a brainstem satiety centre. In natural and laboratory settings, animals have distinct times of the day or circadian phases at which they prefer to eat, but if and how circadian signals affect DVC activity is not well understood. Here, we evaluate how intrinsic circadian signals regulate molecular and cellular activity in the area postrema (AP), nucleus of the solitary tract (NTS), and dorsal motor nucleus of the vagus (DMV) of the DVC. The hierarchy and potential interactions among these oscillators and their response to changes in diet are considered a simple framework in which to model these oscillators and their interactions is suggested. We propose possible functions of the DVC in the circadian control of feeding behaviour and speculate on future research directions including the translational value of knowledge of intrinsic circadian timekeeping the brainstem.

## Introduction

The rotation of the Earth on its axis results in prominent recurring 24 h changes in environmental conditions, which constitute considerable evolutionary pressure on all life on our planet. To anticipate and adjust to these cyclical events, living organisms evolved intrinsic timekeeping mechanisms “ticking” with a period of around 24 h, named circadian clocks. In mammals, the suprachiasmatic nucleus of the hypothalamus (SCN) is considered as the master circadian clock, with its molecular and electrical activity governing most 24 h rhythms in physiology and behaviour. Individual cells of the SCN function as autonomous circadian oscillators. This endogenous circadian timekeeping is possible due to their expression of so-called “core clock genes” which compose a transcriptional-translational feedback loop (TTFL) in which transcription of clock genes is regulated by their own protein products (Takahashi, 2017; Hastings et al., 2018; Hastings et al., 2019). However, discoveries over the past 20 years highlight that cells in several other brain structures rhythmically express clock genes even when completely isolated from the SCN (Abe et al., 2002; Guilding and Piggins, 2007; Begemann et al., 2020; Paul et al., 2020). Thus, it is now accepted that at least some circadian control of physiology is devolved to a network of autonomous and semi-autonomous extra-SCN oscillators (Guilding and Piggins, 2007).

To date, most research on the extended neural circadian system has focused on and identified forebrain structures as potential extra-SCN oscillators. For example, discontinuous sampling of clock gene and/or protein expression *in vivo* demonstrates that limbic and hypothalamic structures rhythmically express components of the molecular clock (Verwey and Amir, 2009; Harbour et al., 2013). In the case of the extended amygdala, lesioning of the SCN or adrenalectomy abolishes these rhythms, indicating that they are dependent on recurrent input from both the SCN and circulating corticosterone (Lamont et al., 2005; Segall et al., 2009). This range of temporal influences highlights the challenge in determining the autonomy of an extra-SCN brain oscillator *in situ*. The development of animals in which the molecular clock is reported by luciferase production [such as the PERIOD2::LUCIFERASE (PER2::LUC) mouse; Yoo et al., 2004] and subsequent continuous monitoring of this bioluminescent signal in cultured brain slices allows such limitations to be circumvented. Results from several studies demonstrate that rhythmic PER2::LUC expression is sustained for several days in distinct structures including the olfactory bulb (Granados-Fuentes et al., 2004a; Granados-Fuentes et al., 2004b), mediobasal hypothalamus (Guilding et al., 2009), thalamus (Chrobok et al., 2021b), and epithalamus (Guilding et al., 2010; Baño-Otálora and Piggins, 2017). These sites can sustain such rhythms when completely disconnected from the SCN and isolated from daily variation in hormone levels, body temperature, etc. Of note, unusually sustained rhythms persisting for weeks in culture were recently revealed in the brain’s sensory circumventricular organs (CVOs). These structures lack a functional blood-brain barrier and this enables them to continuously monitor peripheral signals circulating in the blood (Northeast et al., 2020). The significance of robust circadian timekeeping at the interface of the brain and periphery is yet to be determined but could allow these structures to gate if and when they and surrounding brain areas respond to blood-borne signals.

A key property of SCN neurons is that the TTFL drives them to express circadian rhythms in excitability, such that they discharge action potentials at a higher rate during the day and lower firing rate at night, and this can be monitored both *in vivo* and *in vitro* (Brown and Piggins, 2007; Colwell, 2011). This electrical signalling facilitates synchrony among SCN clock cells as well as communication of time-of-day information to the rest of the brain. The extent to which extra-SCN brain oscillators also vary neuronal activity is underexplored, but findings from the olfactory bulb, mediobasal hypothalamus, and epithalamus indicate that daily rhythms in neuronal activity persist in these structures *in vitro* (Granados-Fuentes et al., 2004b; Guilding et al., 2009; Sakhi et al., 2014). Clock-driven neuronal rhythms in the SCN are also accompanied by pronounced daily rhythms in intracellular calcium, being higher during the day than the night (Belle et al., 2014; Enoki et al., 2017; Hastings et al., 2018). As with electrical rhythms, daily variation in intracellular calcium is not extensively studied in extra-SCN brain oscillators.

Circadian timekeeping in the SCN is not the sole preserve of neurons as astroglia in this structure also exhibit rhythms in intracellular calcium and glutamate uptake (Hastings et al., 2017; Brancaccio et al., 2019). In extra-SCN brain sites including the choroid plexus of the ventricles, non-neuronal cells including astrocytes and ependymocytes also possess circadian timekeeping properties (Guilding et al., 2010; Myung et al., 2018; Chrobok et al., 2020). Through co-culturing experiments and genetic silencing of clock genes in defined cellular subpopulations, it is now recognized that these non-neuronal cells can use long range signals to unidirectionally adjust the circadian period of neuronal populations. For example, the choroid plexus is distal to the SCN, but circadian oscillators in this tissue signal non-synaptically to alter the period of the SCN (Myung et al., 2018). Thus, interaction among neuronal and non-neuronal cells in different brain regions shapes circadian patterns in physiological and behavioural processes.

Such a distributed network of brain oscillators could enable the circadian system to be flexible and responsive to a diverse range of stimuli. Environmental light is the dominant entraining cue or Zeitgeber for the SCN clock (Brown, 2016), but other circadian oscillators are activated and entrained by different Zeitgebers. For example, restricting the daily opportunity to eat to the early day phase is a potent synchroniser of nocturnal rodent behavioural rhythms, particularly in the absence of light (photic) cues (Fuller et al., 2008; Verwey and Amir, 2009; Mistlberger, 2011). This is manifested as a predictable, recurring increase in exploratory behaviour or food anticipatory activity (FAA) approximately 2–3 h in advance of the daily meal. Expression of FAA can be evoked by restricted feeding in both SCN intact and SCN-lesioned animals. Consequently, this SCN independent rhythmic behaviour is interpreted as evidence for a food entrainable oscillator (FEO). Intriguingly, localising the anatomical locus of the FEO has proven exceedingly difficult and no one brain site or peripheral tissue appears to be essential for FAA. This raises the possibility that the FEO is composed of a network of interacting functionally redundant oscillators. Further, in rodents, long-term administration of the psychostimulant methamphetamine elicits >24 h behavioural rhythms (Honma et al., 1987). Similar to the FEO, the anatomical substrates of this so-called methamphetamine sensitive circadian oscillator or MASCO remain unknown but notably, it can be evoked in SCN-lesioned animals (Honma et al., 1988). It remains to be determined if there are shared components or substrates of the FEO and the MASCO, but the evidence clearly shows that they are both independent of the SCN.

Identifying the neuroanatomical sites of extra-SCN oscillators is one step to understanding the extended neural circadian system but there are several gaps in our knowledge to be addressed and among the most prescient concerns are the possible mechanisms and potential routes of communication across this network of oscillators. The SCN directly innervates relatively few brain structures, and its neuronal efferents synapse within only a subset of known extra-SCN brain oscillators (Watts et al., 1987; Kalsbeek et al., 2006; Morin, 2012). Additionally, signals on the circadian timescale are unlikely to be communicated by the means of fast synaptic transmission. By contrast, slower peptidergic signalling is well suited for long-lasting modulation of neuronal activity. Thus volume transmission is a likely candidate to mediate phase across neuroanatomical distal brain oscillators (van den Pol, 2012). This possibility is supported by evidence for humoral signalling between non-neuronal and neuronal oscillators (Myung et al., 2018).

To date, most studies of the properties and functions of extra-SCN brain oscillators have concerned structures in the forebrain and only recently in midbrain (Chrobok et al., 2021a), and consequently knowledge of potential circadian timekeepers in the more caudal structures is impoverished. The aim of this perspective is to provide an overview of recent findings of potential autonomous circadian timekeeping in the hindbrain. Specifically, we evaluate evidence for clock gene rhythms, consider the potential cellular basis of circadian timekeeping (non-neuronal vs. neuronal) and role of its independent components, and discuss possible routes of their communication with each other as well as other brain timekeeping centres. Further, we present a brief perspective of modelling these oscillators and functional considerations, to attempt to position the hindbrain clock in the FEO framework.

## Circadian timekeeping in the dorsal vagal complex

The dorsal vagal complex (DVC) is a multicomponent brainstem centre processing metabolic, cardiovascular, and other homeostatic information and has extensive connectivity with both higher brain structures as well as peripheral organs (Grill and Hayes, 2012). Three independent neuron-containing structures are readily distinguishable in the DVC: the area postrema (AP), nucleus of the solitary tract (NTS), and dorsal motor nucleus of the vagus nerve (DMV). These are positioned adjacent and suprajacent to the ependymal cell layer lining the walls of the central canal/4th ventricle.

The AP is the only sensory CVO in the hindbrain and is implicated in nausea, osmoregulation, and drinking behaviour (Miller and Leslie, 1994; McKinley et al., 2003; Ryan, 2018). More recently it has become clear that AP neurons are also involved in the inhibition of food intake (Roth et al., 2010; Abegg et al., 2017). Similarly, a subset of NTS neurons positioned immediately subjacent to the AP are implicated in signalling satiety. These NTS neurons are responsive to many metabolically relevant peptide signals including ghrelin, cholecystokinin, orexin, leptin, and glucagon-like peptides (Grill and Hayes, 2012; Chen et al., 2020). Additionally, the NTS is a primary receiver of the vagal afferent input from the gastro-intestinal tract. The activation of this pathway results in meal termination. Recently, it has been demonstrated that by receiving these gut-brain information from so called neuropod cells the NTS forms an integral part of the gut-induced reward pathway (Han et al., 2018; Kaelberer et al., 2018; Bai et al., 2019; Chen et al., 2020). Therefore, the NTS acts as the integrator and processor of the DVC, collating vagal information with signals from the AP, hypothalamus, and periphery. This information is further relayed onto the DMV, whose axons form the vagal efferent nerve (Browning and Travagli, 2014; Travagli and Anselmi, 2016).

Non-neuronal and neuronal populations of the DVC are also responsive to other signals. For example, the proximity of DVC structures to the 4th ventricle enables them to detect and process chemical cues circulating in the cerebro-spinal fluid. Further as the AP lacks a blood-brain barrier, neurons within the AP can directly access blood-borne chemical signals (McKinley et al., 2003) and convey this information by synaptic connections to the NTS. Finally, tanycyte-like atypical ependymocytes (recently named “vagliocytes”) form a specialised glial barrier between the AP and NTS. The permeability of this barrier is plastic and serves to regulate the entry of blood-borne cues into the brain parenchyma (Wang et al., 2008; Maolood and Meister, 2009; Langlet et al., 2013; Troadec et al., 2022). Thus, in contrast to the SCN for which light is a key external sensory cue, sensory cues of the internal milieu are of principal relevance to cell populations of the DVC.

**FIGURE 1:**
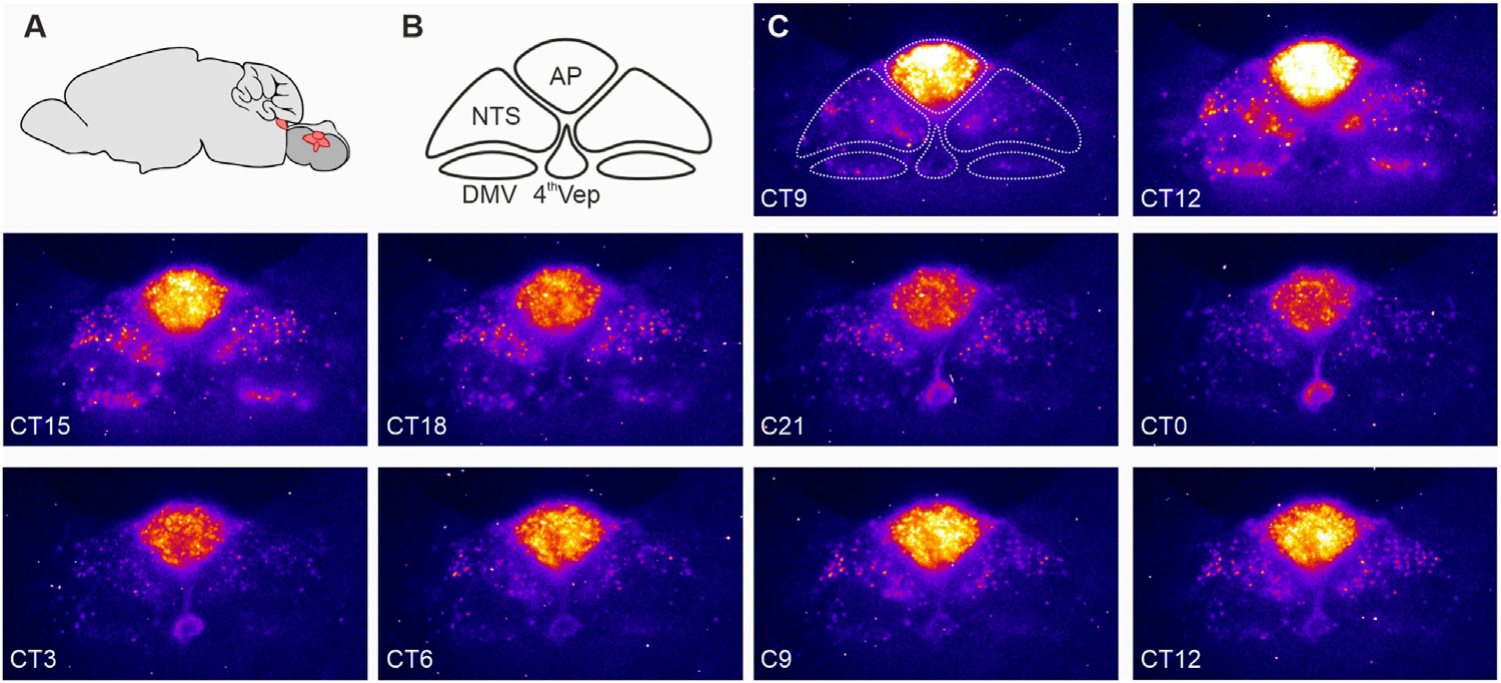
PERIOD2::LUCIFERASE (PER2::LUC) expression in the dorsal vagal complex (DVC). **(A)** Schematic representation of the mouse brain with the brainstem (red) sectioned coronally at the level of the DVC and rotated 90° to illustrate the anatomical location of the DVC. **(B)** Components of the DVC: the area postrema (AP), nucleus of the solitary tract (NTS), dorsal motor nucleus of the vagus nerve (DMV), and ependymal cells lining the fourth ventricle (4thVep). **(C)** Raw bioluminescence images with the DVC structures depicted in B overlayed the first image. Coronal brain slices (250 um) were collected from 10 to 20 week old male PER2::LUC mice and cultured in an Olympus Luminoview LV200 (Olympus, Japan) equipped with a cooled Hamamatsu ImageEM C900-13 EM-CCD camera. Bright colours code high PER2::LUC expression. Note how the intensity and distribution of the bioluminescent signal changes between each consecutive images taken every 3 h. Unpublished example from Chrobok et al., 2020.

Recently, we observed that the DVC exhibits unusually robust and hierarchically organized autonomous circadian timekeeping properties (Chrobok et al., 2020; Chrobok et al., 2021c). Single cell oscillations in PER2::LUC expression were most synchronised and sustained in the AP, with rhythmic cells in the NTS prone to desynchronise more quickly while those in the DMV only transiently (∼1 day) expressed PER2::LUC. Interestingly, ependymal cells lining the wall of the central canal/4th ventricle exhibited robust circadian oscillations in PER2::LUC expression which peaked in near antiphase to those of AP (Chrobok et al., 2020). This resembles PER2::LUC rhythms in the hypothalamic ependymal cells of the 3rd ventricle which also tend to be in antiphase of PER2::LUC rhythms of neurons in the adjacent arcuate and dorsomedial nuclei (Guilding et al., 2009). These findings of rhythmic PER2 expression in DVC slices *ex vivo* were confirmed *in vivo*–both the AP and NTS maintained appropriately-phased rhythmic expression of core clock genes, such as *Per2* and *Bmal1* (Chrobok et al., 2020). Thus, this evidence points to local neuronal and non-neuronal circadian timekeeping in the hindbrain centre (Figure 1).

Similar to the SCN and mediobasal hypothalamus, these molecular rhythms were accompanied with daily variation in DVC neuronal activity. In DVC brain slices disconnected from the SCN, action potential firing rate in the AP and NTS was increased over the day, reaching its peak at the late day/early night phase. Thus, peak neuronal activity in the DVC occurs 5–7 h after the zenith in SCN electrical activity (Brown and Piggins, 2007; Colwell, 2011). Intriguingly at the transition from day-to-night, the AP-NTS glial barrier increased its permeability so that blood-borne molecules were able to penetrate the NTS. In contrast, at the early day phase, the permeability of this barrier was much reduced. This temporal change in permeability is potentially explained by a significant down-regulation in the expression of tight-junction proteins at the early night phase (Chrobok et al., 2020). Thus, the molecular clock activities in the DVC likely drive pronounced daily variation in neuronal activity and access of NTS neurons to blood-borne information.

It remains unresolved if or how the SCN communicates with DVC structures. Findings from neuroanatomical tract-tracing studies indicate that SCN efferents are absent or sparse in the DVC region, which argues against a prominent role for direct synaptic signalling of circadian phase information from the SCN (Buijs et al., 2003; Kalsbeek et al., 2004; Kalsbeek et al., 2006). However, as the SCN is positioned close to the 3rd ventricle, volume transmission of circadian cues in the cerebro-spinal fluid remains a possibility. Obvious candidates for such SCN-derived signals include the peptides prokineticin-2 and vasopressin. The synthesis of these peptides is under circadian control and their receptors are heavily expressed in the DVC (Cheng et al., 2002; Zhou and Cheng, 2005; Bittman, 2009; Li et al., 2009). Outwith the SCN, the DVC forms dense reciprocal connections with several hypothalamic sites, including those implicated in the control of food intake such as the dorsomedial, paraventricular, and lateral hypothalamic nuclei (Shapiro and Miselis, 1985; Peyron et al., 1998; Buijs et al., 2003; Chen et al., 2020). As dorsomedial and paraventricular nuclei are potential extra-SCN oscillators, it is tempting to speculate that circadian information regarding hunger and satiety state is exchanged between the DVC and hypothalamus to shape the daily pattern of ingestion.

## Dependency on diet–the hindbrain clock as a part of food-entrainable oscillator network

Knowledge of circadian timekeeping properties of DVC structures is not matched by understanding of their physiological roles. Some insight can be gleaned by considering the neuroanatomical divisions of the DVC at which clock gene expression can be monitored. For example, cells of the NTS that rhythmically express PER2::LUC are localised exclusively to the intermediate level of the complex at which the AP is also contained, and are absent at rostral and caudal levels of the NTS where the AP is not present (Chrobok et al., 2020). Since this intermediate division of the DVC is implicated in metabolic function and not processing of cardiovascular (caudal) or oral sensation (rostral part) cues (Grill and Hayes, 2009; Grill and Hayes, 2012), then it follows that these DVC timekeepers may regulate the times of day at which ghrelin and stomach distension activate DVC neurons (Sabbatini et al., 2004; Carneiro and Araujo, 2009). However, they are unlikely to be part of the FEO network since FAA persists in rats with AP and NTS lesions (Davidson et al., 2001).

Nonetheless, timekeeping in the DVC is overtly responsive to changes in diet. For example, long-term consumption of a high-fat diet (HFD) blunts the daily profile in expression of core clock genes in the mouse NTS *in vivo* (Kaneko et al., 2009). Recently, we also determined similar effects of HFD on neuronal activity in the rat DVC (Chrobok et al., 2022a; Chrobok et al., 2022b). We found that even short-term consumption of HFD (3–4 weeks) elevated daytime feeding and flattened the typical day-to-night increase in feeding. Further, this HFD dampened or eliminated day-to-night variation in parameters of DVC neuronal activity, including basal firing rate and responsiveness to metabolically relevant peptides (Chrobok et al., 2022a; Chrobok et al., 2022b). Unexpectedly, the late day peak in neuronal activity in the NTS was completely eliminated in HFD-fed rats. This may contribute to the altered daily pattern of food intake seen in these HFD animals by diminishing the signalling of satiety by these NTS neurons (Chrobok et al., 2022b). However, it is still unclear if the DVC clock is directly affected by the constituents of the diet itself or by feedback from the altered daily feeding pattern observed in rodents fed HFD (Engin, 2017; Chrobok et al., 2022b). Further studies are needed to distinguish these possibilities.

## Spatiotemporal organisation of clock gene expression in the dorsal vagal complex

Clock cells in the SCN are well-synchronised, but they do not all achieve peak *Per2* expression at exactly the same time/phase. Instead, the SCN exhibits an apparent wave of ordered phases in clock gene expression throughout its dorso-ventral axis. Current evidence indicates that the plasticity in phase distribution and phase gap between the dorsal and ventral SCN enable the circadian clock in the SCN to code daylength and thereby potentially act as a seasonal calendar (Meijer et al., 2010; Coomans et al., 2015; Myung and Pauls, 2018). Therefore, phase differences between tissue and cellular clocks may code sensory information and be of physiological relevance.

Similar to the master SCN clock, phase distribution of clock gene expression in the DVC is not homogenous (Chrobok et al., 2020). PER2::LUC bioluminescence recordings *ex vivo* revealed a pronounced sequence in core clock gene expression, with the AP leading the rhythm, the NTS following, and the 4th ventricle ependymal layer peaking in antiphase with the AP (Figure 1). This phase arrangement stabilized for several days in culture and was reinitiated following an initial strong synchronisation of the DVC oscillators with forskolin. These phase gaps are potentially governed by intrinsic connectivity between the AP and NTS, and the AP and the 4th ventricle oscillator. Such functional connectivity is seen when electrically stimulating and recording the AP and NTS (Hay and Bishop, 1991; Chrobok et al., 2020; Chrobok et al., 2021c). In mouse, the AP is a unilateral structure located along the midline of nervous system, whereas the NTS is bilateral, with an NTS each on the left and the right side of the brainstem. When one NTS is physically disconnected from the AP, its circadian period in PER2::LUC oscillations accelerates, whereas the connected NTS in the same tissue slice maintains a longer circadian period. The period of PER2::LUC rhythms in the AP remains unperturbed when surgically disconnecting it from one of the NTSs (Chrobok et al., 2020). This indicates that the AP circadian oscillator is more resilient than the NTS circadian oscillator and suggests that an AP-derived signal acts to regulate the circadian period of the NTS.

Computational models can be used to understand how phase gaps appear between synchronised biological oscillators (Myung and Pauls, 2018; Schmal et al., 2019). One approach is to conceptualise the DVC as a network of Kuramoto-like oscillators. This can be used to simulate how the AP can synchronise the NTS through unidirectional efferents. The same conceptual framework can be used to model if and how the AP communicates its circadian phase to the ependymal layer. In this simulation, the AP acts as the main oscillator. At present the physiological relevance of AP-ependyma interaction is unclear, but a modelling approach could be used to guide future experiments and to pinpoint functions of the phase distribution of circadian oscillations in the DVC (Gonze, 2011; Hastings et al., 2017; Myung and Pauls, 2018; Chrobok et al., 2020).

## Summary and perspective

In summary, there is considerable evidence for a network of circadian oscillators within defined structures of the brainstem DVC. Electrophysiological recordings indicate that molecular clock oscillations drive neuronal rhythms in the AP and NTS, while assessment of the AP-NTS glial barrier reveals that it is more permeable at the transition to circadian night. This is coincident with reduced expression of genes for tight junction components. Elevation of dietary content in fat dampens day-night changes in the molecular and electrophysiological activity of the DVC. Although the precise physiological roles of the DVC circadian oscillators remain to be determined, these findings raise the possibility that they are responsive to diet constituents and regulate daily access of blood-borne signals to the brain. Thus, their contribution to daily patterns in ingestive behaviour should be of prime focus in future investigations.

**FIGURE 2:**
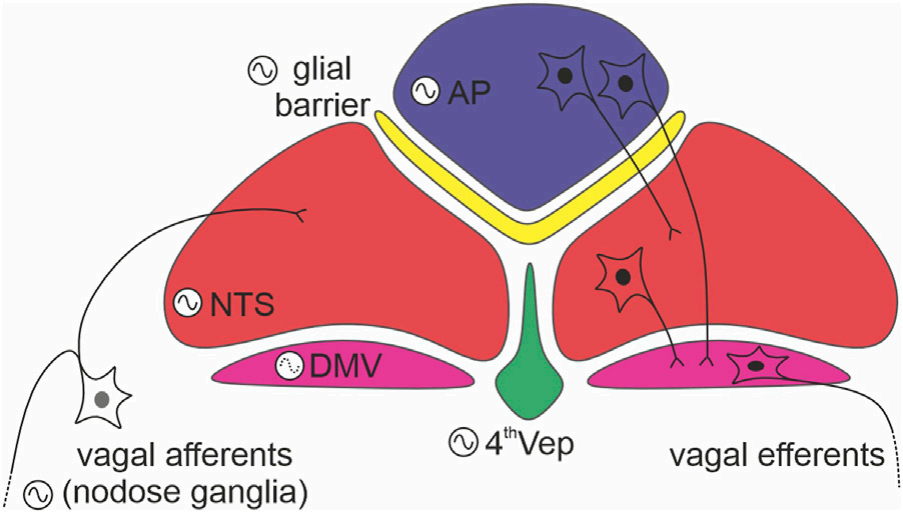
Internal connections between dorsal vagal complex (DVC) oscillators. A schema depicting readily defined components of the DVC: the area postrema (AP; in blue), glial barrier (in yellow), nucleus of the solitary tract (NTS; in red), dorsal motor nucleus of the vagus nerve (DMV; in purple), and ependymal layer lining the fourth ventricle (4thVep; in green). Neurons indicate the main direction of synaptic communication amongst the oscillators. Note, that the DMV (forming vagal efferents) is classified as a weak oscillator (dotted line in its oscillation logo).

Since DVC structures exhibit pronounced phase order in their time of peak oscillations, then a key goal of future should be to determine if and what is measured by these phase gaps. Further, establishing how the AP regulates the NTS as well as the ependyma oscillations will be important to guide future studies of the physiological relevance of DVC circadian timekeeping. Fundamental to this research will be to identify the cellular (neuronal vs. non-neuronal) and neurochemical phenotype of cells that function as bona fide clock cells in the DVC. In this regard, the utilization of recently published RNAseq datasets from the DVC will be of particular interest (Alkaslasi et al., 2021; Dowsett et al., 2021). Further, modelling this network of anatomically separate though proximal oscillators should inform understanding of oscillator interactions in general (Figure 2).

## Data Availability

The original contributions presented in the study are included in the article/supplementary material, further inquiries can be directed to the corresponding authors.
